# Flowery ln_2_MnSe_4_ Novel Electrocatalyst Developed via Anion Exchange Strategy for Efficient Water Splitting

**DOI:** 10.3390/nano12132209

**Published:** 2022-06-28

**Authors:** Sumaira Manzoor, Sergei V. Trukhanov, Mohammad Numair Ansari, Muhammad Abdullah, Atalah Alruwaili, Alex V. Trukhanov, Mayeen Uddin Khandaker, Abubakr M. Idris, Karam S. El-Nasser, Taha AbdelMohaymen Taha

**Affiliations:** 1 Institute of Chemical Sciences, Bahauddin Zakariya University, Multan 60800, Pakistan; mnumairansari96@gmail.com; 2 Laboratory of Magnetic Films Physics, SSPA “Scientific and Practical Materials Research Centre of NAS of Belarus”, 19, P. Brovki Str., 220072 Minsk, Belarus; truhanov86@mail.ru; 3 Department of Chemistry, Government College University, Lahore 54000, Pakistan; abdullah843gcu@yahoo.com; 4 Physics Department, College of Science and Arts, Jouf University, Al-Gurayyat P.O. Box 756, Saudi Arabia; abdoanas161@gmail.com; 5 Laboratory of Single Crystal Growth, South Ural State University, 76, Lenin Av., 454080 Chelyabinsk, Russia; 6 Department of Electronic Materials Technology, National University of Science and Technology MISiS, 119049 Moscow, Russia; 7 Centre for Applied Physics and Radiation Technologies, School of Engineering and Technology, Sunway University, Bandar Sunway 47500, Malaysia; mayeenk@sunway.edu.my; 8 Department of General Educational Development, Faculty of Science and Information Technology, Daffodil International University, DIU Rd, Dhaka 1341, Bangladesh; 9 Department of Chemistry, College of Science, King Khalid University, Abha 62529, Saudi Arabia; 10 Research Center for Advanced Materials Science (RCAMS), King Khalid University, Abha 62529, Saudi Arabia; 11 Chemistry Department, College of Science and Arts, Jouf University, Al-Gurayyat P.O. Box 756, Saudi Arabia; karamsaif@ju.edu.sa; 12 Chemistry Department, Faculty of Science, Al-Azhar University, Assiut 71524, Egypt; 13 Physics Department, College of Science, Jouf University, Sakaka P.O. Box 2014, Saudi Arabia; taha.hemida@yahoo.com; 14 Physics and Engineering Mathematics Department, Faculty of Electronic Engineering, Menoufia University, Menouf 32952, Egypt

**Keywords:** In_2_MnSe_4_, flower shape, anion exchange method, electrocatalyst, water splitting

## Abstract

Oxygen and hydrogen generated by water electrolysis may be utilized as a clean chemical fuel with high gravimetric energy density and energy conversion efficiency. The hydrogen fuel will be the alternative to traditional fossil fuels in the future, which are near to exhaustion and cause pollution. In the present study, flowery-shaped In_2_MnSe_4_ nanoelectrocatalyst is fabricated by anion exchange reaction directly grown on nickel foam (NF) in 1.0 M KOH medium for oxygen evolution reaction (OER). The physiochemical and electrical characterization techniques are used to investigate the chemical structure, morphology, and electrical properties of the In_2_MnSe_4_ material. The electrochemical result indicates that synthesized material exhibits a smaller value of Tafel slope (86 mV/dec), lower overpotential (259 mV), and high stability for 37 h with small deterioration in the current density for a long time. Hence, the fabricated material responds with an extraordinary performance for the OER process and for many other applications in the future.

## 1. Introduction

Global energy consumption will continue to increase in the near future, owing to the rise in population and industry. However, conventional non-renewable fossil fuels such as coal, gas, and oil have so many adverse effects on the environment and living beings, so it is vital that ecologically friendly and renewable energy sources be discovered and used in place of fossil fuel [1,2]. The consumption of fossil fuels such as coal and petroleum has fully poisoned the atmosphere with pollutants such as carbon monoxide and nitrogen oxide, etc. As a result of these emissions, the earth’s temperature rises, pollution increases, and water sources become more acidic [3,4]. The depletion of non-renewable fossil fuels, as well as the negative environmental impacts of fossil fuel use, has prompted extensive research into breakthrough technology for finding sustainable energy resources, such as wind and solar, in order to save the environment as well as human lives. Wind and solar are both plentiful renewable energy resources and they can be transformed into electrical energy, but both are weather-dependent and irregular in terms of their space requirements [5,6,7]. Researchers are focused on establishing resources for the development of unstable electrical energy into stable chemical energy such as H_2_, which can act as potential fuel with zero carbon emissions and can replace traditional fossil fuel. However, water splitting is a novel method and a risk-free way of manufacturing hydrogen and is the method with the most potential for producing a sustainable energy substitute in the short-term.

Energy is used in water splitting to split water molecules as a half-cell reaction, such as hydrogen evolution reaction (HER) at the cathode and oxygen evolution reaction (OER) at the anode [8]. In particular, electrochemical water splitting is utilized to generate non-polluted energy at lower cost in three distinct mediums, such as acidic, neutral, and alkaline [9]. Usually, the OER process mostly occurs in a basic medium and the reaction mechanism is given as 2OH^−^ + H_2_O ↔ ½O_2_ + 2e^−^. A 1.23 V standard potential (E) in the presence of a reversible hydrogen electrode (RHE) with a thermodynamic Gibbs free energy (G) of 237.2 kJ mol^−1^ is required for water splitting.

The two-electron transfer (HER) and four-electron–proton-coupled reaction (OER) create enormous energy barriers which significantly slow down the electrocatalytic water-splitting kinetics [10,11]. Therefore, researchers are interested in developing such electrocatalysts, which are required to accelerate the slow OER kinetics by lowering their kinetic energy barriers and, as a result, significantly enhancing the production of H_2_ energy efficiency. Because of their long-term stability and excellent efficiency, iridium-based electrodes are frequently utilized as electrode materials for water oxidation reactions [12]. These noble-metal-based catalysts are rarely employed at the industrial level due to their scarcity and expensive cost. However, various materials are employed in place of noble metals that have specific alignment, shape, physical and chemical properties [13]. Transition metals and their derivatives exhibited better properties than noble metals, and they could replace the expensive and rare earth metals [14,15,16].

Catalysts made of metal complexes, such as Fe [17], Ni [18,19], Mn [20], Zn [21], Co [22], and Cd [23] have been utilized as electrocatalysts, photocatalysts, or both. The characteristics and activity of these transition metals can be increased in several ways resulting in excellent OER results, which has increased the level of research interest in electrocatalysis [24]. While transition metal oxides Al_2_O_3_ [25] BiVO_4_ [26], SnO_2_ [27,28,29], sulfides [30], carbides [31], nitrides [32], selenides [33], and tellurides [34,35] have significantly increased electrocatalytic capabilities when combined with other metals, the overpotential and onset potential to achieve a state-of-the-art current is constantly decreasing due to their high surface area, electronic structure, morphology and higher catalytic site [36].

Among all candidates, the metal selenide (M-Se) such as ZnSe [37], CuSe [38], NiSe_2_ [39] and CdSe [40] have good potential because they possess an outer electron in d-subshell, and the metallic and electrical properties of Se at the transition metal edge contain the immoderate active site which significantly increases the efficiency of these materials. Moreover, enormous active sites and the tailoring of the surface area led to an enhancement in the electrochemical properties of the Se-based electrocatalyst. Owing to a contradiction between activity and stability, it is impossible to combine two independent electrodes with different properties in the same system in a new design modification. As a result, it is critical to create nanostructured materials utilizing a surface technique that has superior characteristics [41].

From the aforementioned characteristics, in this study, we prepared a novel In_2_MnSe_4_ nano electrocatalyst for oxygen evolution reaction fabricated by anion exchange reaction. The various complementary physiochemical techniques were employed to investigate the structure, morphology and electrical properties of fabricated In_2_MnSe_4_. For electrochemical characterizations, the electrocatalyst-loaded electrode was grown on the nickel foam (NF) substrate via the drop cast method, and the porous, highly conductive loaded 3D- nickel foam substrate is responsible for demonstrating such outstanding results with long-term stability.

## 2. Experimental Segment

### 2.1. Reagents

Indium nitrate hexahydrate (In(NO_3_)_3_. 6H_2_O, Sigma-Aldrich/99.99%, Luoyang Tongrun Nano Technology Co. Ltd., Luoyang, China), manganese nitrate tetra hydrate, (Mn(NO_3_)_2_.4H_2_O, Sigma-Aldrich/99.99%, Luoyang Tongrun Nano Technology Co. Ltd., Luoyang, China), sodium nitrate (NaNO_3,_ Sigma Aldrich/99.99%, Luoyang Tongrun Nano Technology Co. Ltd., Luoyang, China), Selenium powder (Sigma-Aldrich/99%, Luoyang Tongrun Nano Technology Co. Ltd., Luoyang, China), ammonium fluoride (NH_4_F-Merck/98%, Luoyang Tongrun Nano Technology Co. Ltd., Luoyang, China), H_2_O_2_ (Sigma-Aldrich, 30%), sodium borohydride (NaBH_4_-Sigma-Aldrich, 98%) were used as such without any further treatment.

### 2.2. Fabrication of In_2_MnSe_4_

In_2_MnSe_4_, the electrocatalyst was prepared by a typical anion exchange reaction. For this purpose, the whole procedure was divided into two steps, firstly, InMn-LDH (layered double hydroxides) nanosheets were prepared by preparing solutions of 0.05 M Mn(NO_3_)_2_.4H_2_O, 0.05 M of In(NO_3_)_3_.6H_2_O, 0.08 M of NaNO_3_ and 0.1 M of NH_4_F in 125 mL of N_2_-saturated deionized water. After that, 20 μL of hydrogen peroxide (H_2_O_2_) was poured, and then 40 mL of N_2_ saturated NaOH solution (0.08 M) was added slowly while stirring on the magnetic hotplate. The reaction was maintained at room temperature for 15 h under a N_2_ atmosphere. InMn-LDH nanosheets were collected and dried under vacuum conditions. Secondly, 5 mg of the synthesized LDH nanosheets and 8.2 mg of Se particles were dissolved in 20 mL of deionized water, and then 10 mg of NaBH_4_ under stirring conditions. After 15 min of the stirring, the mixed solutions were then poured into hydrothermal reactor which was set at 190 °C for 15 h. Then, the autoclave was cooled down to room temperature, and the precipitates were collected by filtration and rinsed with deionized water multiple times. Finally, these precipitates were dried and stored in air-sealed vials for further processing.

### 2.3. Characterizations

X-ray diffraction (XRD) in the range of 20–90° employing a Cu K radiation source (=1.5406) was used to assess the phase identification and lattice parameters of the developed nano electrode material. The surface morphology and elemental makeup of In2MnSe4 were explored using a scanning electron microscope (SEM) coupled with energy dispersive X-Ray spectroscopy (EDX) (Nano Nova SEMa-450). The functional group on the surface of the materials was confirmed via Fourier transform infrared spectroscopy (FTIR) using Alpha FTIR spectrometer, and the Brunauer Emmett Teller (BET) method measured the specific surface area of the as-synthesized material using the Micromeritics ASAP-2020 instrument.

### 2.4. Ink Preparation and Electrochemical Measurements

In this study, the In_2_MnSe_4_ working electrode was fabricated hydrothermally and grown on the NF substate via the drop cast method. For this purpose, the NF was divided into 1 × 1 cm pieces and washed. For ink preparation in water, isopropanol and 10 µL of Nafyon (5 weight percent) were mixed to make a dispersed solution after ultrasonification for 1 h. Catalyst ink was applied on the clean and already-treated NF substrate by the drop-casting method and dried at ambient temperature before being used. Three electrode electrochemical experiments were performed using a PGSTAT (AUTOLAB-204) electrochemical workstation (Metrohm, Herisau, Switzerland), having a platinum wire, Ag/AgCl electrode, and the fabricated electrode act as a counter, reference, and working electrode, respectively. Various complementary electrochemical techniques were used; the measurements included chronoamperometry (CA), linear scan voltammetry (LSV), electrochemical impedance spectroscopy (EIS), cyclic voltammetry (CV), and electrochemical active surface area (ECSA). The CV was carried out at a scan rate of 5 mV s^−1^, with both positive and negative potentials utilized in the cyclic voltametric experiments, while only the positive potential was used in the LSV. Equation (1) was used to calculate the *RHE* potential from the *Ag/AgCl* potential:

(1) $$ERHE=EAg/AgCl+0.059pH+E_{0\;}Ag/AgCl$$

The overpotential was determined by using the Formula (2):

(2) $$Overpotential=E_{RHE}-1.23\;V$$

From 1000 kHz to 1 Hz, electrochemical impedance measurements were carried out utilizing 5 mV sinusoidal voltages. The Faradaic efficiency (FE) was calculated using the total charge (*Q*) and total oxygen generation (*O*_2_) (*nO*_2_) with the following Equation (3):

(3) $$F.E=4F{nO}_{2}/Q$$

The polarization curvature on a Tafel graph was used to monitor the catalytic and kinetics of the fabricated material with the following Equation (4):

η = a + (2.303RT/αnF) × log j(4)

Here, η, α, n, j and F are corresponding overpotentials, charge transfer coefficient, number of e^-^ taking part in the reaction, current density, and Faraday constant, respectively. Equation (5) was used to calculate the TOF value of In_2_MnSe_4_ coated on nickel foam for the OER process.

TOF = I/4 × F × m(5)

Here, m = number of moles of catalytic material, I = current in amperes, and F = Faraday constant.

The surface area of the catalyst was measured by using cyclic voltammetry recording at different scan rates in the non-faradic region in order to measure the electrical double layer capacitance (Cdl) by plotting the change in current density vs. scan rate. The resultant Cdl value has a direct relation with the electrochemical active surface area. The electrochemical impedance approach was used in alkaline solution vs. *Ag/AgCl* at a voltage of 0.5 V in the frequency range of 0.01–100 kHz. The Rct and Rs values were calculated using the NOVA 2.1 software via PGSTAT-204(Metrohm, Herisau, Switzerland).

## 3. Results and Discussion

### 3.1. Structural Analysis

**Figure 1:**
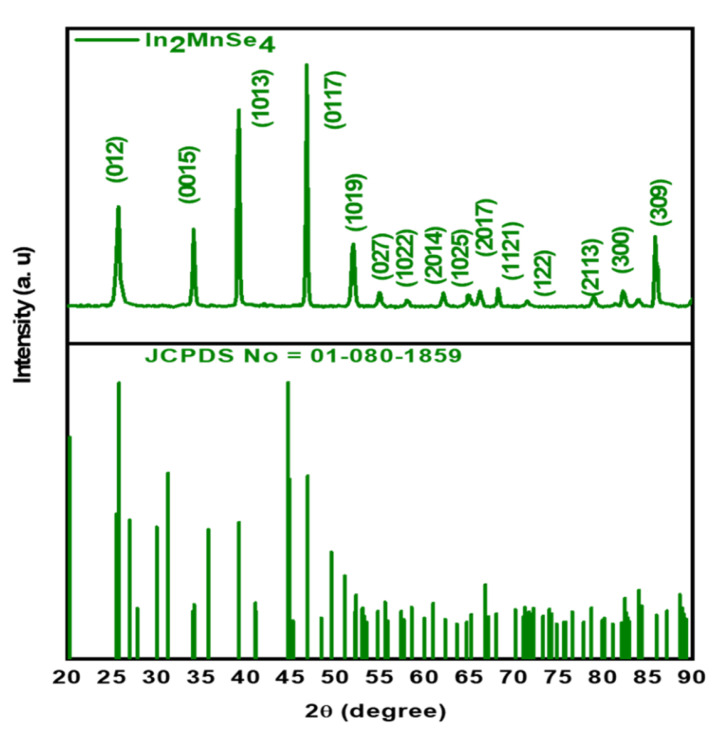
XRD diffractogram of the fabricated In_2_MnSe_4_ nanostructure.

The crystal structure, geometry, purity, and orientation of as-prepared indium manganese selenide were analyzed by executing XRD characterization as shown in Figure 1. The diffraction peaks of In_2_MnSe_4_ appeared at 2θ = 25.6⁰, 33.9⁰, 39.2⁰, 46.7⁰, 52⁰, 54.8⁰, 57.9⁰, 61.9⁰, 64.8⁰, 66.1⁰, 68.2⁰, 71.5⁰, 78.8⁰, 82.2⁰, 83.8⁰, and 85.9⁰, and can be indexed to the planes (012), (0015), (1013), (0117), (1019), (027), (1022), (2014), (1025), (2017), (1121), (122), (2113), (300), and (309), respectively. The resultant planes were accurately matched with JCPS card no. 01-080-1859. The sharp and well-defined XRD pattern of the prepared structure and absence of the secondary peaks confirms the pure crystalline phase of the prepared material. The crystal planes thus obtained correspond to the rhombohedral nanostructure with cell constant values of a = 4.051 Å, b = 4.051 Å, and c = 39.464 Å. The following expression was utilized to measure the crystallite size of the In_2_MnSe_4_ nanomaterial using the Debye Scherrer Equation (6) [42].

(6) D=Kλ/β cos θ 

where *D* represents average crystallite size, *K* constant (0.9), *λ* is wavelength of X-ray, *β* depicts line broadening at FWHM and *θ* = Bragg’s angle. The calculated crystalline size of the synthesized particle using Scherrer’s formula was 68.3 nm.

**Figure 2:**
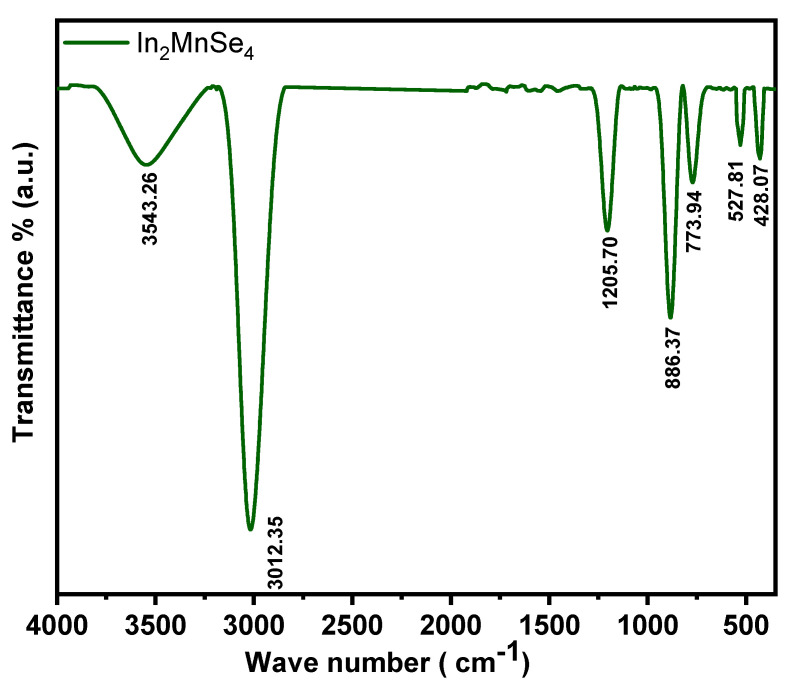
FTIR analysis of In_2_MnSe_4_ nanostructure.

FTIR analysis was employed to investigate the purity and functional groups of the synthesized nanomaterials. The observed spectrum also confirmed the existence of metal in FTIR spectrum of In_2_MnSe_4_ nanomaterials (400–4000 cm^−1^) as shown in Figure 2. From the spectra, the characteristic peaks of vibrational bands positioned at 428.07 cm^−1^, 527.81 cm^−1^, and 773.94 cm^−1^ correspond to the Mn-Se, In-Se, and Se-Mn-Se bond vibrations, respectively, corroborating the effective synthesis of the In_2_MnSe_4_ nanomaterials, while the additional peak located at 1205.70 cm^−1^ was due to N-O stretching due to the presence of NH_4_F.

### 3.2. Morphological, Elemental, and Textural Analysis

**Figure 3:**
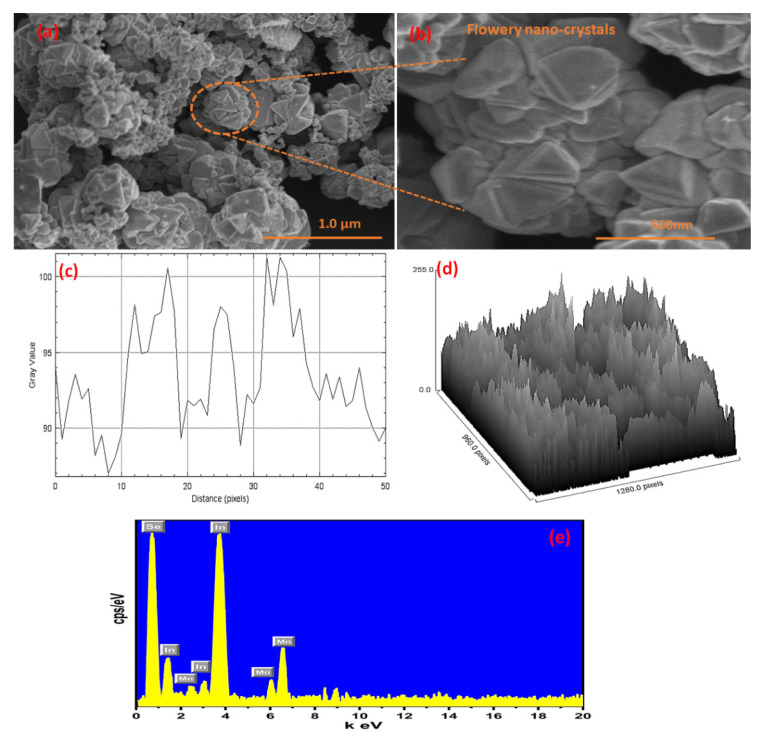
(**a**,**b**) SEM micrograph, (**c**) particle size calculated from SEM micrograph, and (**d**) Surface plot, and (**e**) EDX spectrum of the synthesized In_2_MnSe_4_ nanostructure.

The surface dimension, shape, and particle size of the produced material were investigated using scanning electron microscopy (SEM). SEM micrographs indicates that the material exhibited a flowery shape with a definite crystalline boundary as shown in Figure 3a,b. These flowery nanocrystals are responsible for plenty of electrochemically active sites and facilitate the redox reactions at the electrode, thus showing significantly outstanding electrochemical performances for OER. The crystallite size of the fabricated nanoparticle was 83.28 nm, calculated from an SEM micrograph by IMAGE.J software as shown in Figure 3c,d using plot profile and surface plot.

The elemental compositions were studied by using energy-dispersive X-ray spectroscopy (EDS) analysis as shown in Figure 3e. The examination reveals that the synthesized products include all of the individual elements, such as In, Mn, and Se, that are consistently aligned as shown in Figure 3e. The obtained results show that the synthesized product has no additional interfering components.

**Figure 4:**
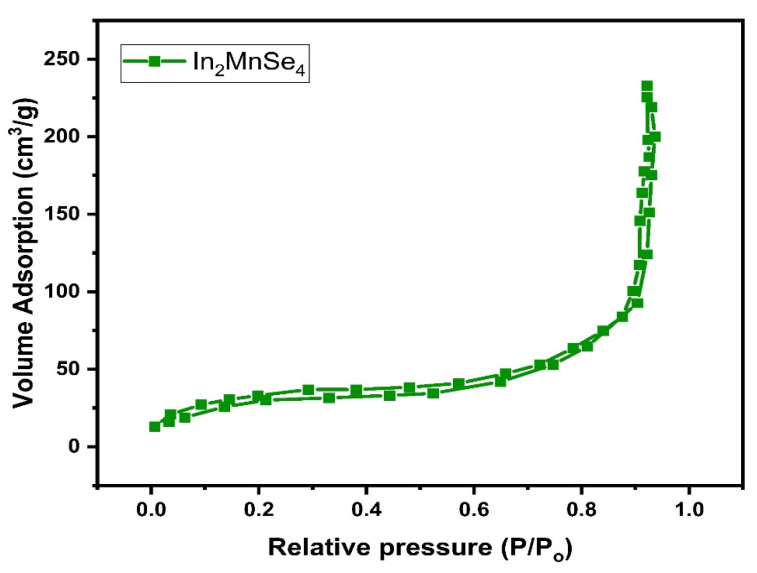
BET isotherm of the synthesized In_2_MnSe_4_ nanostructure.

A nitrogen adsorption–desorption experiment was also used to investigate the surface attributes of the nanostructure, such as specific surface area (SSA) and average pore size as shown in Figure 4. The surface area of the In_2_MnSe_4_ was 30 m^2^ g^−1^ with a pore size and pore volume of 0.002 Å, and 0.25 cm^3^ g^−1^, respectively. A high surface area stimulates the formation of more vacant sites on the surface of the fabricated catalyst. As a result, the obtained material was a suitable electrocatalyst for the oxygen evolution reaction (OER).

### 3.3. Electrical Measurement

**Figure 5:**
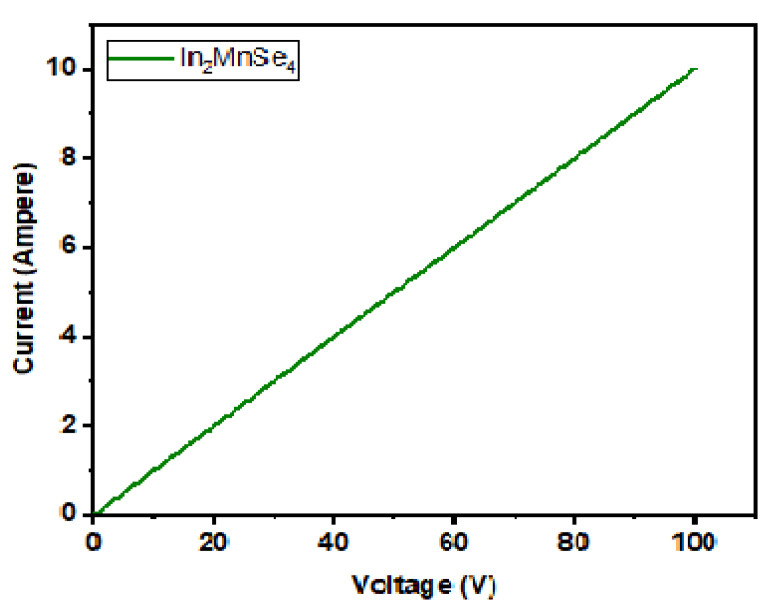
I–V polarization curve of In_2_MnSe nanostructure.

I–V curves of synthetic products with potential ranges of 0–20 V were measured at ambient temperature. For terminal voltages, the graph clearly shows that the nanomaterials exhibited a higher current value. The ohmic quality of the produced nanocomposite was also proven by the curve. The electrical conductivity (3.1 × 10^2^ moh) of the forward biased region was calculated using the following Equation (7):

(7) σ=I×L/V×A

where I = current, V = applied voltage, L = the thickness of the pellet, and A = the cross-sectional area of the pellets. The variation as a function of current concerning the voltage is seen in Figure 5. The grown nanostructure performs an excellent choice for OER because of its ohmic nature.

### 3.4. Electrochemical Study

The porous flowery-shaped In_2_MnSe_4_ nanostructure has been used for the first time for an oxygen evolution reaction. A three-electrode configuration was used to test the loaded NF as a working electrode for the oxygen evolution reaction in an alkaline solution at room temperature. Electrodes were categorized into two categories based on their structure such as flat surface electrodes and 3D electrodes. The flat-surface electrodes, such as glassy carbon (GC) substrates and indium-doped tin oxide (ITO) substrates, as well as Cu/Ti foil, allow for single-way electrolyte penetration, controlling the reaction on the surface of the catalyst due to the non-porous nature. Furthermore, the 3D electrode integrates all active species in the catalytic process, such as carbon cloth/paper (CC/CP) and Ni foam (NF), and allows electrolytes to permeate in all dimensions via various pathways.

The most used electrode, for example, is the GC/GP electrode, which indicates the catalyst’s initial activity. Among all the options, the NF demonstrates high electrochemical performance due to its high conductivity, and the performance of the nickel foam working electrode has a significant impact on the rate of reaction due to its degree of wettability, structure, electrolyte, and conductivity. The loading of the catalyst on the nickel foam was kept constant for the uniformity during dipping the electrode in 8 mL electrolyte, and the geometrical surface area (0.5 cm^2^) of the nickel foam electrode was taken into account while measuring the current density.

**Figure 6:**
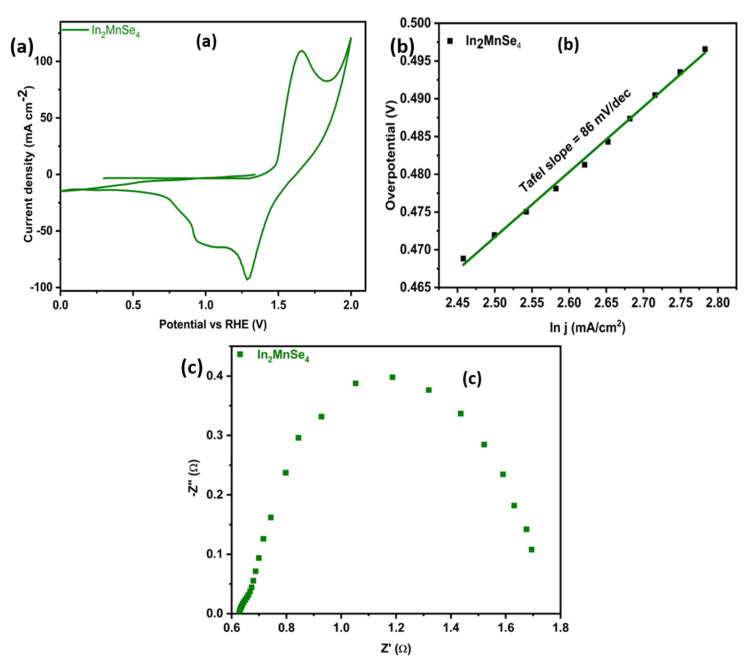
(**a**) Cyclic voltammogram (CV), (**b**) Tafel slope of In_2_MnSe_4_ nan, and (**c**) EIS IR Corrected Nyquist plot for In_2_MnSe_4_.

The porous nature, high electrical conductivity and good morphology of the catalyst species boosted OER activity, in terms of the Tafel slope, onset potential, overpotential, ECSA, and stability. Cyclic voltammetry was carried out using a stationary electrode in 1.0 M KOH electrolyte at room temperature, saturated with nitrogen, in a potential range (1 to −1 vs. Ag/AgCl), with a scan rate of 5 mV s^−1^ to achieve an overpotential of 259 mV to attain a current density of 10 mA cm^−2^. A typical cyclic voltammogram for In_2_MnSe_4_ is shown in Figure 6a.

Figure 6b displays the Tafel slope of In_2_MnSe_4_ to confirm the kinetics of the reaction, and the calculated Tafel slope was found to be 86 mV/dec as represented as in Figure 6b. The turnover frequency was another important parameter to investigate the OER performance of the fabricated material, and it shows a direct relation with OER performance, i.e., the higher the TOF, the higher the OER activity will be. The calculated TOF of the In_2_MnSe_4_ was 0.00348 s^−1^. The subsequent results were compared with the already reported results as presented in Table 1.

Electrochemical impedance spectroscopy was also employed to study the electrochemical catalytic features of fabricated material, as shown in Figure 6c. The high-frequency range showed the solution resistance (Rs) at the semicircle’s origin, the mid-frequency range showed the charge transfer resistance (Rct) from the semicircle’s diameter, and the low-frequency range showed the ion diffusive zone as a vertical line. The resultant Rs and Rct values are 12.73 and 1.20 ohms, respectively, according to the Nyquist impedance plot as shown in Figure 6c, indicating the high conductivity of the designed material.

**Figure 7:**
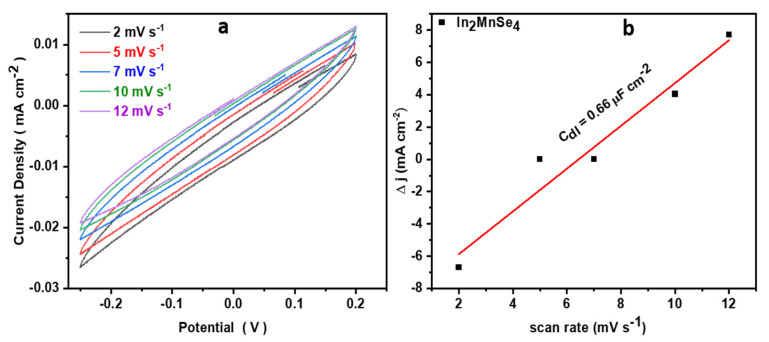
Electrochemical active surface area test (ECSA) (**a**) CV data with multiple scan rate and (**b**) electrochemical double layer capacity (C_dl_) of In_2_MnSe_4_.

In addition to the non-Faradaic zone, an electrochemical active surface area (ECSA) study was investigated using CV curves at different scan rates between 0 and 0.1 V vs. Ag/AgCl, as shown in Figure 7a. The resultant electrical double layer capacitance of 0.66 mF calculated by plotting the scan rate vs. change in the current density. The obtained double layer capacitance was used to calculate the ECSA of In_2_MnSe_4_, and was found to be 16.65 cm^2^, considering the specific capacific capacitance of the 0.04 mF cm^−2^ using equation ECSA = Cdl/Cs (specific capacitance).

**Figure 8:**
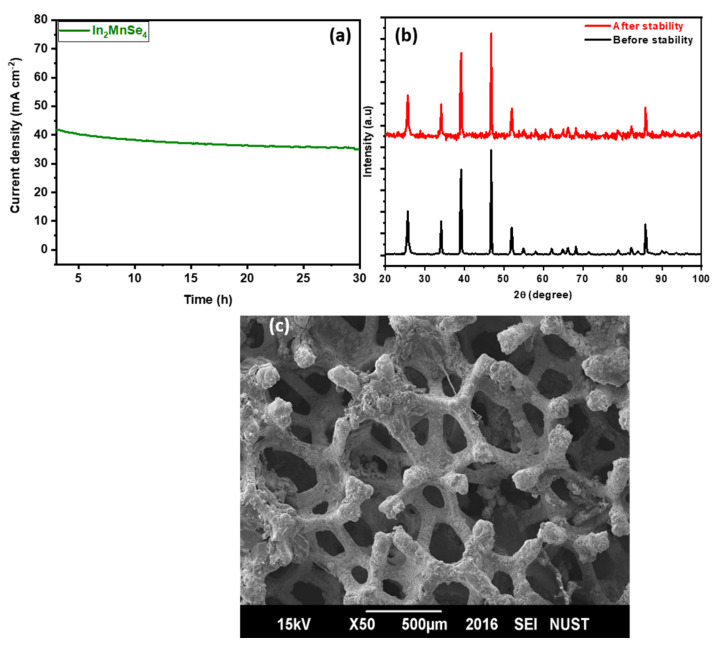
(**a**) I–t curve measurement for ln_2_MnSe_4_ nanostructure, XRD pattern, SEM micrograph before and after stability. (**b**) XRD patterns of samples before and after stability. (**c**) SEM foto of ln_2_MnSe_4_ nanostructure.

In addition, the newly synthesized ln_2_MnSe_4_ catalyst showed substantial OER stability. The chronoamperometry was employed to measure the working electrodes for long-term operational catalytic activity, and the results revealed that it was stable for 30 h with no noticeable decline in current density values, attributed to the electrode’s high stability. This demonstrates potential for practical use in water electrolysis as shown in Figure 8. To prove the structure of the synthesized material, XRD and SEM were performed, which confirmed that the structure and morphology remained same after 30 h of stability as shown in Figure 8b,c. Hence, the enhanced electrochemical performance of the synthesized materials was due to the good flower-shaped surface morphology, because the resultant flower-shaped morphology provides more active sites on the surface of the fabricated material as confirmed via SEM micrographs, high surface area, and high conductivity as indicated via BET and I–V curves, respectively.

## 4. Conclusions

In this study, a facile and one-pot hydrothermal manufacturing technique was employed to produce a novel, economical nanostructured metal selenide (In_2_MnSe_4_), and was characterized by different analytical techniques. For electrochemical measurements, the fabricated material was deposited on Ni foam that functions as a high-performance oxygen evolution reaction. The fabricated In_2_MnSe_4_ electrode has the lowest overpotential of 259 mV with a smaller Tafel slope value of 86 mV dec^−1^, and has the long-term durability of 30 h. Our research has shown novel methodologies for generating and fabricating In_2_MnSe_4_ nanoarchitecture that have low-cost, high-efficiency, and customizable catalysts for clean fuel generation.

## Figures and Tables

**Table 1:** Comparison of various OER parameters of the present material with already reported materials.

Sr. No.	Material Name	Overpotential mV	Tafel mV dec^−1^	Electrolyte	Electrode Type	Ref.
1	Sm_2_O_3_/Fe_2_O_3_	272	75	1.0M KOH	Graphite Pencil	[29]
2	Fe dopedNi_2_S_3_/rGO	247	63	1.0M KOH	Ni-foam	[43]
3	Co-S/Ti-mesh	361	64	1.0M KOH	Graphite	[44]
4	CoO_x_ film	403	42	1.0 MKOH	Glass electrode	[45]
5	MnFeSe	247	35	1.0 MKOH	Ni foam	[46]
6	NiCo LDH	367	40	1.0 MKOH	Carbon paper	[47]
7	Fe/Ni-BTC@NF	270	47	0.1M KOH	Ni-foam	[48]
8	Co_2_P nanoneedles	310	50	1.0M KOH	Glassy Carbon	[49]
9	Cd(OH)_2_	266	47	1.0M KOH	Ni-foam	[50]
10	MAFX27-OH	387	60	1.0M KOH	Glassy Carbon	[51]
11	Pb-TCPP	470	106	1.0M KOH	Glassy Carbon	[52]
12	ZnCoTe	221	91	1.0M KOH	Graphite pencil	[53]
13	Mn-Cd-S@Ni_3_S_2_	333	150	1.0M KOH	Ni-foam	[54]
**14**	**In_2_MnSe_4_**	**259**	**86**	**1.0M KOH**	**NF**	**Present work**

## Data Availability

Not applicable.

## References

[1] Stambouli A.B. (2011). Fuel cells: The expectations for an environmental-friendly and sustainable source of energy. Renew. Sust. Energy Rev..

[2] Lin Z., Xiao B., Huang M., Yan L., Wang Z., Huang Y., Shen S., Zhang Q., Gu L., Zhong W. (2022). Realizing negatively charged metal atoms through controllable d-electron transfer in ternary Ir_1−x_Rh_x_Sb intermetallic alloy for hydrogen evolution reaction. Adv. Energy Mater..

[3] Yang L., Yang L., Ding L., Deng F., Luo X.-B., Luo S.L. (2019). Principles for the application of nanomaterials in environmental pollution control and resource reutilization. Nanomaterials for the Removal of Pollutants and Resource Reutilization, Micro and Nano Technolgies.

[4] Shen S., Hu Z., Zhang H., Song K., Wang Z., Lin Z., Zhang Q., Gu L., Zhong W. (2022). Highly active Si sites enabled by negative valent Ru for electrocatalytic hydrogen evolution in LaRuSi. Angew. Chem..

[5] Opoku F., Govender K.K., van Sittert C.G.C.E., Govender P.P. (2018). Tuning the electronic structures, work functions, optical properties and stability of bifunctional hybrid graphene oxide/V–doped NaNbO_3_ type–II heterostructures: A promising photocatalyst for H_2_ production. Carbon.

[6] Hu E., Ning J., He B., Li Z., Zheng C., Zhong Y., Zhang Z., Hu Y. (2017). Unusual formation of tetragonal microstructures from nitrogen-doped carbon nanocapsules with cobalt nanocores as a bi-functional oxygen electrocatalyst. J. Mater. Chem. A.

[7] Zhang Q., Guan J. (2021). Applications of atomically dispersed oxygen reduction catalysts in fuel cells and zinc–air batteries. Energy Environ. Mater..

[8] Najafi L., Oropesa-Nuñez R., Bellani S., Martín-García B., Pasquale L., Serri M., Drago F., Luxa J., Sofer Z.K., Sedmidubský D. (2022). Topochemical transformation of two-dimensional VSe_2_ into metallic nonlayered VO_2_ for water splitting reactions in acidic and alkaline media. ACS Nano.

[9] Tang T., Wang Z., Guan J. (2022). A review of defect engineering in two-dimensional materials for electrocatalytic hydrogen evolution reaction. Chin. J. Catal..

[10] Seh Z., Kibsgaard J., Dickens C., Chorkendorff I.B., Nørskov J.K., Jaramillo T.F. (2017). Combining theory and experiment in electrocatalysis: Insights into materials design. Science.

[11] Morales-Guio C.G., Stern L.-A., Hu X. (2014). Nanostructured hydrotreating catalysts for electrochemical hydrogen evolution. Chem. Soc. Rev..

[12] Zhang Q., Guan J. (2022). Applications of single-atom catalysts. Nano Res..

[13] Li Z., Lu X., Teng J., Zhou Y., Zhuang W. (2021). Nonmetal-doping of noble metal-based catalysts for electrocatalysis. Nanoscale.

[14] Chen H., Yan J., Wu H., Zhang Y., Liu S. (2016). One-pot fabrication of NiFe_2_O_4_ nanoparticles on α-Ni(OH)_2_ nanosheet for enhanced water oxidation. J. Power Sources.

[15] Zhu P., Xiong X., Wang D. (2022). Regulations of active moiety in single atom catalysts for electrochemical hydrogen evolution reaction. Nano Res..

[16] Fan K., Ji Y., Zou H., Zhang J., Zhu B., Chen H., Daniel Q., Luo Y., Yu J., Sun L. (2017). Hollow iron–vanadium composite spheres: A highly efficient iron-based water oxidation electrocatalyst without the need for nickel or cobalt. Angew. Chem. Int. Ed..

[17] Bai X., Wang L., Nan B., Tang T., Niu X., Guan J. (2022). Atomic manganese coordinated to nitrogen and sulfur for oxygen evolution. Nano Res..

[18] Tian T., Huang L., Ai L., Jiang J. (2017). Surface anion-rich NiS_2_ hollow microspheres derived from metal–organic frameworks as a robust electrocatalyst for the hydrogen evolution reaction. J. Mater. Chem. A.

[19] Kuang P., Tong T., Fan K., Yu J. (2017). In situ fabrication of Ni–Mo bimetal sulfide hybrid as an efficient electrocatalyst for hydrogen evolution over a wide pH range. ACS Catal..

[20] Xu D., Xia T., Xu H., Fan W., Shi W. (2020). Synthesis of ternary spinel MCo_2_O_4_ (M = Mn, Zn)/BiVO_4_ photoelectrodes for photolectrochemical water splitting. Chem. Eng. J..

[21] Digraskar R.V., Sapner V.S., Mali S.M., Narwade S.S., Ghule A.V., Sathe B.R. (2019). CZTS decorated on graphene oxide as an efficient electrocatalyst for high-performance hydrogen evolution reaction. ACS Omega.

[22] Wang J., Cui W., Liu Q., Xing Z., Asiri A.M., Sun X. (2016). Recent progress in cobalt-based heterogeneous catalysts for electrochemical water splitting. Adv. Mater..

[23] Yang X., Zhou Y., He J. (2020). Two unexplored two-dimensional MSe_2_ (M = Cd, Zn) structures as the photocatalysts of water splitting and the enhancement of their performances by strain. Vacuum.

[24] Weng B., Xu F., Wang C., Meng W., Grice C.R., Yan Y. (2017). A layered Na_1−x_Ni_y_Fe_1−y_O_2_ double oxide oxygen evolution reaction electrocatalyst for highly efficient water-splitting. Energy Environ. Sci..

[25] Ito R., Akatsuka M., Ozawa A., Kato Y., Kawaguchi Y., Yamamoto M., Tanabe T., Yoshida T. (2019). Photocatalytic activity of Ga_2_O_3_ supported on Al_2_O_3_ for water splitting and CO_2_ reduction. ACS Omega.

[26] Jessl S., Rongé J., Copic D., Jones M.A., Martens J., De Volder M. (2019). Honeycomb-shaped carbon nanotube supports for BiVO_4_ based solar water splitting. Nanoscale.

[27] Chen S.-Y., Yang J.-S., Wu J.-J. (2018). Three-dimensional undoped crystalline SnO_2_ nanodendrite arrays enable efficient charge separation in BiVO_4_/SnO_2_ heterojunction photoanodes for photoelectrochemical water splitting. ACS Appl. Energy Mater..

[28] Ghosh K., Zhang W., Tassinari F., Mastai Y., Lidor-Shalev O., Naaman R., Moöllers P., Nuürenberg D., Zacharias H., Wei J. (2019). Controlling chemical selectivity in electrocatalysis with chiral CuO-coated electrodes. J. Phys. Chem..

[29] Abid A.G., Manzoor S., Usman M., Munawar T., Nisa M.U., Iqbal F., Ashiq M.N., Najam-ul-Haq M., Shah A., Imran M. (2021). Scalable synthesis of Sm_2_O_3_/Fe_2_O_3_ hierarchical oxygen vacancy-based gyroid-inspired morphology: With enhanced electrocatalytic activity for oxygen evolution performance. Energy Fuel..

[30] Sumesh C.K., Peter S.C. (2019). Two-dimensional semiconductor transition metal based chalcogenide based heterostructures for water splitting applications. Dalton Trans..

[31] Volokh M., Peng G., Barrio J., Shalom M. (2019). Carbon nitride materials for water splitting photoelectrochemical cells. Angew. Chem. Int. Ed..

[32] Chen S., Ma G., Wang Q., Sun S., Hisatomi T., Higashi T., Wang Z., Nakabayashi M., Shibata N., Pan Z. (2019). Metal selenide photocatalysts for visible-light-driven Z-scheme pure water splitting. J. Mater. Chem. A.

[33] Sadaqat M., Manzoor S., Nisar L., Hassan A., Tyagi D., Shah J.H., Ashiq M.N., Joya K.S., Alshahrani T., Najam-ul-Haq M. (2021). Iron doped nickel ditelluride hierarchical nanoflakes arrays directly grown on nickel foam as robust electrodes for oxygen evolution reaction. Electrochim. Acta.

[34] Sadaqat M., Nisar L., Hussain F., Ashiq M.N., Shah A., Ehsan M.F., Najam-Ul-Haq M., Joya K. (2019). Zinc-telluride nanospheres as an efficient water oxidation electrocatalyst displaying a low overpotential for oxygen evolution. J. Mater. Chem. A.

[35] Xiong X., You C., Liu Z., Asiri A.M., Sun X. (2018). Co-doped CuO nanoarray: An efficient oxygen evolution reaction electrocatalyst with enhanced activity. ACS Sust. Chem. Eng..

[36] Panda C., Menezes P.W., Zheng M., Orthmann S., Driess M. (2019). In situ formation of nanostructured core–shell Cu_3_N–CuO to promote alkaline water electrolysis. ACS Energy Lett..

[37] Liu E., Zhang X., Xue P., Fan J., Hu X. (2020). Carbon membrane bridged ZnSe and TiO_2_ nanotube arrays: Fabrication and promising application in photoelectrochemical water splitting. Int. J. Hydrogen Energy.

[38] Chakraborty B., Beltrán-Suito R., Hlukhyy V., Schmidt J., Menezes P.W., Driess M. (2020). Crystalline copper selenide as a reliable non-noble electro (pre) catalyst for overall water splitting. ChemSusChem.

[39] Wang P., Pu Z., Li W., Zhu J., Zhang C., Zhao Y., Mu S. (2019). Coupling NiSe_2_-Ni_2_P heterostructure nanowrinkles for highly efficient overall water splitting. J. Catal..

[40] Wu L.L., Wang Q.S., Li J., Long Y., Liu Y., Song S.Y., Zhang H. (2018). Co_9_S_8_ Nanoparticles-embedded N/S-codoped carbon nanofibers derived from metal–organic framework-wrapped CdS nanowires for efficient oxygen evolution reaction. Small.

[41] Wang Y., Kong B., Zhao D., Wang H., Selomulya C. (2017). Strategies for developing transition metal phosphides as heterogeneous electrocatalysts for water splitting. Nano Today.

[42] Kroon R. (2013). Nanoscience and the Scherrer equation versus the’Scherrer-Gottingen equation. S. Afr. J. Sci..

[43] Shao D., Li P., Zhang R., Zhao C., Wang D., Zhao C. (2019). One-step preparation of Fe-doped Ni_3_S_2_/rGO@ NF electrode and its superior OER performances. Int. J. Hydrogen Energy.

[44] Liu T., Liang Y., Liu Q., Sun X., He Y., Asiri A.M. (2015). Electrodeposition of cobalt-sulfide nanosheets film as an efficient electrocatalyst for oxygen evolution reaction. Electrochem. Commun..

[45] Trotochaud L., Ranney J.K., Williams K.N., Boettcher S.W. (2012). Solution-cast metal oxide thin film electrocatalysts for oxygen evolution. J. Amer. Chem. Soc..

[46] Sun M., Gao R.-T., Liu X., Gao R., Wang L. (2020). Manganese-based oxygen evolution catalysts boosting stable solar-driven water splitting: MnSe as an intermetallic phase. J. Mater. Chem. A.

[47] Liang H., Meng F., Cabán-Acevedo M., Li L., Forticaux A., Xiu L., Wang Z., Jin S. (2015). Hydrothermal continuous flow synthesis and exfoliation of NiCo layered double hydroxide nanosheets for enhanced oxygen evolution catalysis. Nano Lett..

[48] Wang L., Wu Y., Cao R., Ren L., Chen M., Feng X., Zhou J., Wang B. (2016). Interfaces, Fe/Ni metal–organic frameworks and their binder-free thin films for efficient oxygen evolution with low overpotential. ACS Appl. Mater. Interf..

[49] Dutta A., Samantara A.K., Dutta S.K., Jena B.K., Pradhan N. (2016). Surface-oxidized dicobalt phosphide nanoneedles as a nonprecious, durable, and efficient OER catalyst. ACS Energy Lett..

[50] Chen X., Wang H., Meng R., Xia B., Ma Z. (2020). Cadmium hydroxide: A missing non-noble metal hydroxide electrocatalyst for the oxygen evolution reaction. ACS Appl. Energy Mater..

[51] Lu X.-F., Liao P.-Q., Wang J.-W., Wu J.-X., Chen X.-W., He C.-T., Zhang J.-P., Li G.-R., Chen X.-M. (2016). An alkaline-stable, metal hydroxide mimicking metal–organic framework for efficient electrocatalytic oxygen evolution. J. Amer. Chem. Soc..

[52] Dai F., Fan W., Bi J., Jiang P., Liu D., Zhang X., Lin H., Gong C., Wang R., Zhang L. (2016). A lead–porphyrin metal–organic framework: Gas adsorption properties and electrocatalytic activity for water oxidation. Dalton Trans..

[53] Majhi K., Karfa P., De S., Madhuri R. (2019). Hydrothermal synthesis of zinc cobalt telluride nanorod towards oxygen evolution reaction (OER). IOP Conf. Ser. Mater. Sci. Eng..

[54] Li Z., Wang X., Wang X., Lin Y., Meng A., Yang L., Li Q. (2019). Mn-Cd-S@ amorphous-Ni_3_S_2_ hybrid catalyst with enhanced photocatalytic property for hydrogen production and electrocatalytic OER. Appl. Surf. Sci..

